# Children’s coordination of the “sweet spot” when striking a forehand is shaped by the equipment used

**DOI:** 10.1038/s41598-020-77627-5

**Published:** 2020-12-03

**Authors:** Tim Buszard, Alessandro Garofolini, David Whiteside, Damian Farrow, Machar Reid

**Affiliations:** 1 Institute for Health and Sport (IHES), Victoria University, Melbourne, Australia; 2 Game Insight Group, Tennis Australia, Melbourne, Australia

**Keywords:** Human behaviour, Motor control, Dynamical systems

## Abstract

Children’s movement coordination is significantly influenced by the equipment used when performing multi-articular actions. Previously we reported that scaled equipment (smaller racket and a softer ball), but not full-sized equipment, promoted a functional coupling between upper arm and forearm angles in children performing a forehand. However, it remains unclear whether the shoulder-racket distance—which is controlled by this coupling—is a performance variable. This study therefore advanced previous research by examining whether the shoulder-racket distance is associated with performance. We also improved our understanding of how the shoulder-racket distance is controlled by including the hand-racket segment in our biomechanical model. Twenty-one children performed 40 forehands in a hitting for accuracy task. Participants were randomly divided into two groups—a scaled equipment group and a full-sized equipment group. Results revealed that the shoulder-racket distance was a performance variable, as evidenced by: (a) its variance reduced closer to ball impact, (b) its distance at ball impact, but not at the start of the forward swing, differentiated good from poor performance, and (c) its distance was similar for both groups, implying that there was a “sweet spot” for striking a ball, regardless of racket size. We also showed that it is the shoulder-racket vector in state-space (i.e., distance and angle) that differentiates good from poor performance. Finally, the manner in which the shoulder-racket distance was controlled differed between the groups, with scaled equipment promoting a more distal control than full-sized equipment. Implications for skill acquisition are discussed.

## Introduction

A central issue for understanding performance of multi-articular actions is how abundant degrees of freedom in the musculoskeletal system are controlled to produce functional movements^1,2^. In tasks that require precise outcomes, such as in sport, the ability to coordinate muscles, joints and limbs to successfully perform the task is complex and challenging. To the naked eye, professional athletes appear to display repeatable and consistent movements, yet variability in movement is an inherent feature of successful performance^3,4^. These seemingly contradictory statements are at the heart of motor control science; that is, to understand how motor control systems constrain the many possible joint configurations to stabilize the salient task-dependent performance variables^5^.

Performance variables (often referred to as *essential variables*) are invariant features of the motor system that can be produced by a number of joint configurations (referred to as *elementary variables*). The elementary variables can either form a synergy, meaning movements at each joint co-vary, or not form a synergy^5^. Synergies are formed by means of *self-organisation* and are governed by the equation of constraints acting on the system^6^. Indeed, the formation of synergies that produce functional outcomes is a hallmark of skilful performance^7^. Understanding how constraints—categorised as task, environmental, and organismic^8^—interact to influence the formation of synergies is therefore essential.

One of the most common constraints in sport is the equipment used by the performer. Differences in size and/or mass change the object’s inertial properties and this subsequently modifies the confluence of constraints^9^. The use of inappropriately sized equipment that leads to qualitative changes in coordination is perhaps most apparent in children’s sport, whereby children are often expected to execute skills with equipment that is designed for adults^10^. For example, we recently reported how different rackets and tennis balls elicited distinct motor control strategies in children^11^. When a full-sized (adult) tennis racket and a standard tennis ball was used, variance in the distance between the shoulder and racket (i.e., the hitting lever) was explained by the angles in the upper arm and forearm, but there was a lack of coupling between these segments. Conversely, when children used a smaller tennis racket and a softer ball, variance in the distance between the shoulder and the racket was explained by the coupling of the upper arm and forearm angles. In other words, these segments worked in unison, whereby variability in one segment was compensated by variability in the other segment. This can be interpreted as the formation of a synergy amongst the elementary variables to control the hitting lever distance. This study was significant as it was the first to investigate the effect of equipment scaling on children’s movement coordination in a multi-articular action.

However, despite the compelling differences in motor control strategies that were observed between scaled and full-sized equipment, at least two issues remain unclear. First, although the upper arm and forearm angles explained the variance in the shoulder-racket distance, we cannot conclude that this distance is a salient performance variable as we did not measure its relationships with performance outcomes. There is a wealth of research on the kinematics of the forehand^12–15^; yet, little is known about the role of the shoulder-racket distance in the coordination of the stroke. Given that this distance determines the lever’s inertial mass, which influences the linear velocity required to strike the ball and generate sufficient force, we expected this distance to be associated with performance—particularly the distance at ball impact as this is the most important moment in striking tasks e.g.^16^. Second, we did not include the hand-racket segment in our model, so we do not know the contribution of this segment in explaining the variance in shoulder-racket distance. Given that the tennis forehand requires proximal to distal sequence of joint rotations^15^, the hand-racket segment is likely to play a central role in explaining the variance in the shoulder-racket distance closer to ball impact.

The aim of the current study was therefore twofold. First, we aimed to determine whether the distance between the shoulder and the racket is a salient performance variable in a tennis forehand task performed by children. We hypothesised that this would be evidenced by (a) a similar shoulder-racket distance across the swing regardless of equipment, therein demonstrating the invariant nature of the variable; (b) performance differences based on shoulder-racket distance at ball impact; and (c) less variance in shoulder-racket distance for more accurate shots. We also measured the distribution of the shoulder-racket vector in state-space based on hitting accuracy. State-space is a 2D plane which was defined by two orthogonal global directions with the origin fixed at the shoulder. We expected the distribution of the shoulder-racket vector to represent a smaller and skinnier ellipse for more accurate shots; hence demonstrating the stability of the shoulder-racket distance for successful performance. For less accurate shots, we expected the distribution of the shoulder-racket vector to be large and structured orthogonal to the high scores. This would suggest that poor performance at the task is associated with a variable shoulder-racket vector in different directions of state-space than successful performance.

The second aim was to assess the contribution of the hand-racket segment in explaining the variance in shoulder-racket distance. We hypothesised that the hand-racket segment would have a greater contribution in explaining the variance in shoulder-racket distance closer to ball impact. We also expected to observe a more even contribution across the three segments (racket, forearm and upper arm) when using scaled equipment than full-sized equipment. This was based on our previous finding whereby scaled equipment promoted co-variation amongst the elementary variables, whereas full-sized equipment did not^11^. Finally, we anticipated that variance in the hand-racket segment angle would play a more significant role explaining variance in the shoulder-racket distance when using scaled equipment, as this is indicative of a finer level of control^17,18^.

## Results

**Figure 1:**
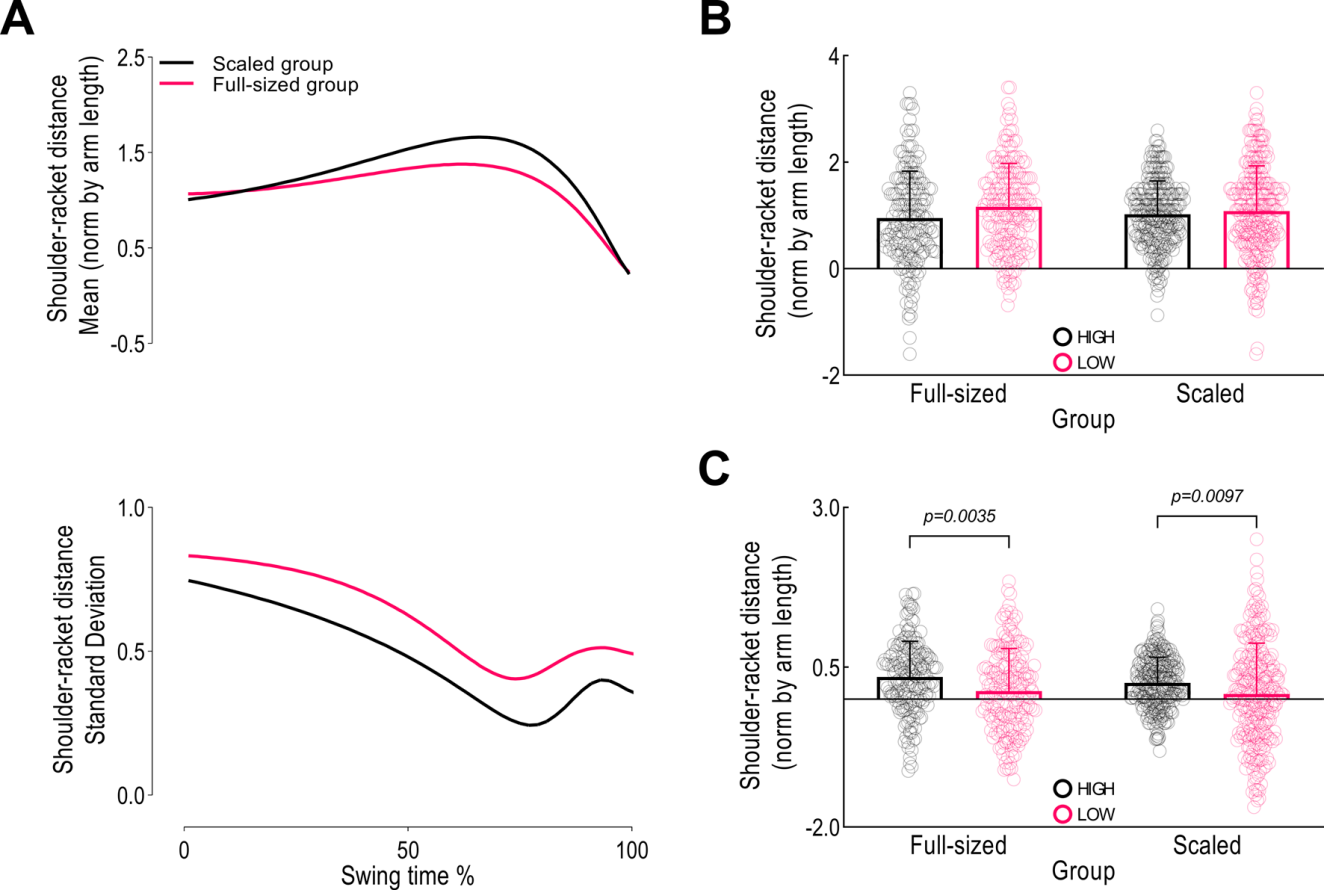
(**A**) Means and standard deviations for shoulder-racket distance across the swing for all participants, and the mean and variance of shoulder-racket distance for high scores (≥ 7) and low scores (≤ 3) at (**B**) the start of the forward swing and (**C**) at ball contact.

Shoulder-racket distance was consistent between groups (Fig. 1A). The scaled racket allowed a longer lever during the swing, but at ball impact both rackets were guided to a distance about 0.3 of arm length. For variability (Fig. 1A), the standard deviation dropped consistently until 75% of the swing time, followed by an increase and a final drop. This was consistent between groups, although the full-sized group displayed higher variability overall.

At the start of the swing, shoulder-racket distance was similar for both low-scoring (*≤ *3, LOW) and high-scoring (*≥ *7, HIGH) trials (Fig. 1B). At impact, there was a main effect for score (F_1,874_ = 21.48, *p* < 0.01) but no main effect for group (F_1,874_ = 2.63, *p* = 0.105). Indeed, both the scaled and full-sized groups had a higher shoulder-racket distance in HIGH compared to LOW scores at ball impact (Fig. 1C).

**Figure 2:**
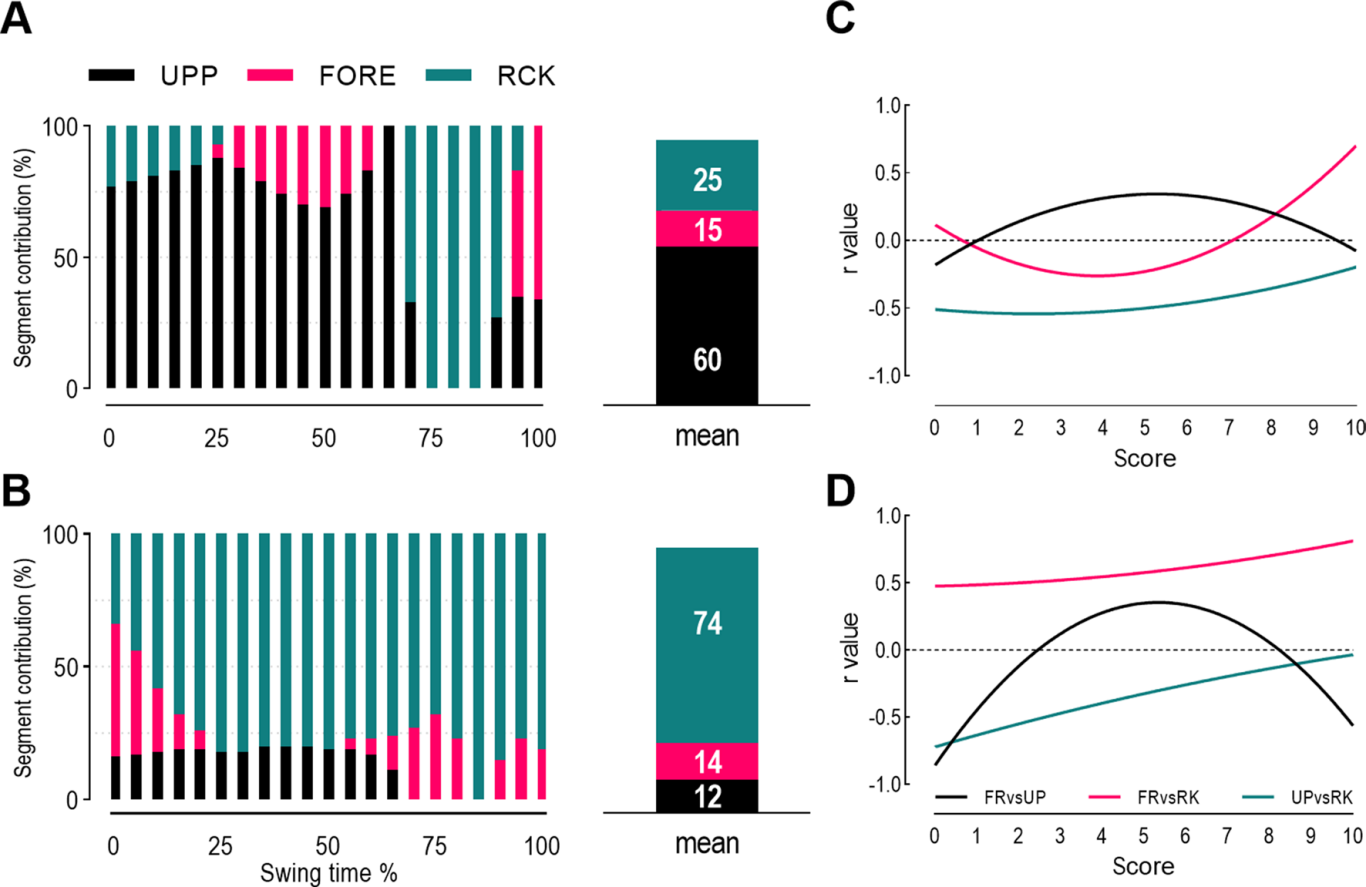
Shoulder-racket distance control contribution of the upper arm (UPP), forearm (FORE), and hand-racket (RCK) segment for full-sized (**A**) and scaled (**B**) equipment, and the second order polynomial fit of correlation values, between the segments’ variance at ball impact across score values for full-sized (**C**) and scaled (**D**) equipment. FR is forearm; UP is upper arm; and RK is racket.

Results from the multiple linear regression showed that the predictors (variance in angles for the upper arm, forearm, and hand-racket segments) significantly explained the variance in shoulder-racket distance in both groups (*p* < 0.01). However, the predictors explained 16.5% of the variance in shoulder-racket distance for the full-sized group, and 27.4% for the scaled group. For the full-sized group, the most important predictor was variance in the upper arm angle (86%) followed by variance in the forearm angle (10%) and variance in the angle of the hand-racket segment (4%). Comparatively, the most important predictor for the scaled group was forearm angle variance (52%), followed by variance in the hand-racket segment’s angle (37%) and variance in the upper arm angle (11%). A closer inspection of the predictors revealed that variance in the upper arm angle contributed most to controlling shoulder-racket distance throughout the swing in the full-sized group (60%). Additionally, the regulatory contributions of the hand-racket segment’s angle only appeared closer to ball impact (Fig. 2A,B). For the scaled racket group, regulatory contributions of the upper arm angle were minimal and the hand-racket segment angle was the main contributor. All the details for the linear multiple regression are reported in Supplementary Tables S1 and S2.

Measurements of segment co-variation (second order polynomial fit) revealed that high scores in both groups were achieved by (a) an increase in negative correlation between variance in the forearm and upper arm angles, (b) an increased correlation between variance in the forearm and hand-racket angles, and (c) an increased (less negative) correlation between variance in the upper arm and hand-racket angles (Fig. 2C,D; model details are reported in Supplementary Tables S3, S4).

**Figure 3:**
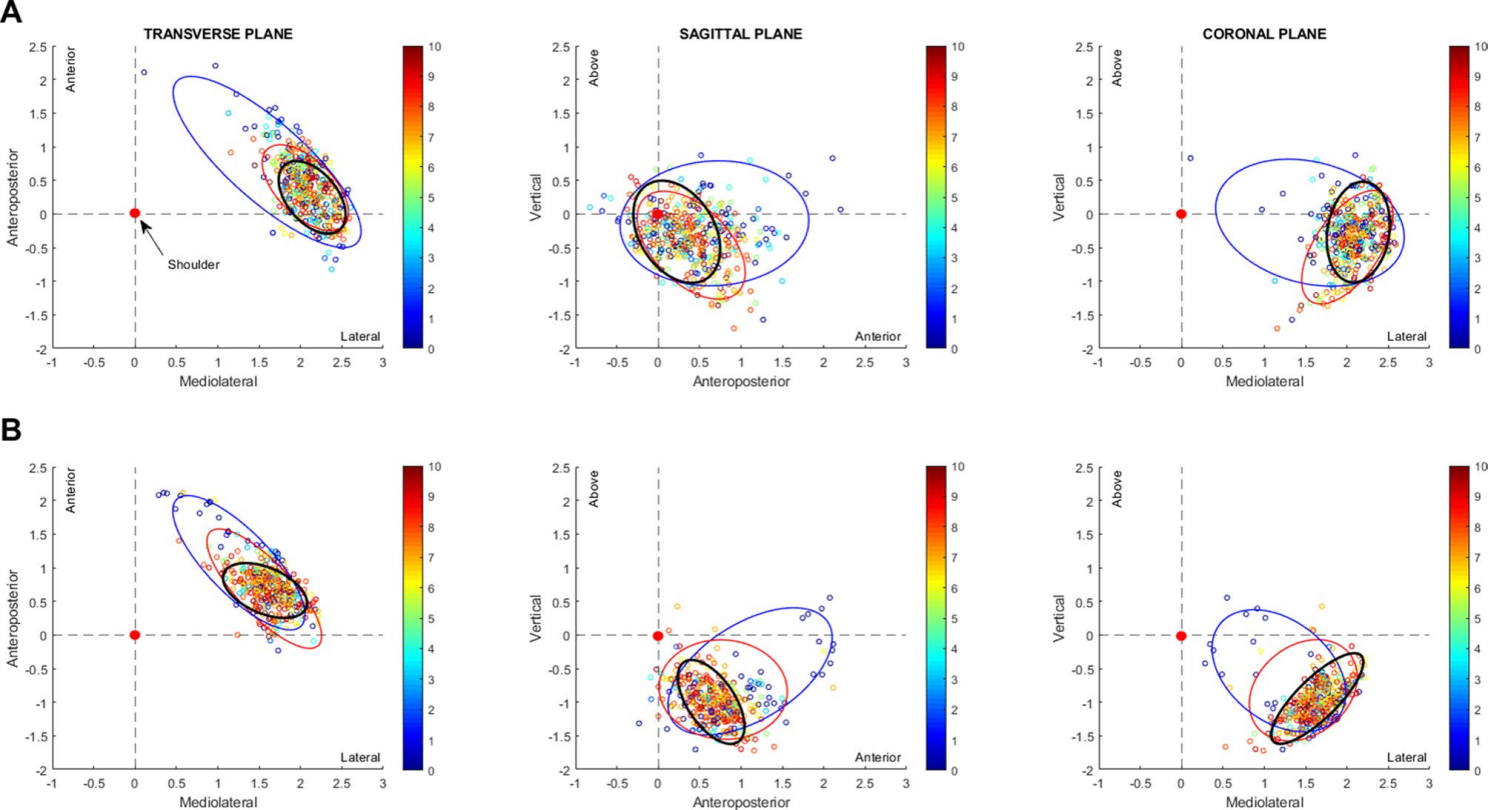
Distribution of shoulder-racket distance at ball impact in the three planes for full-sized (**A**) and scaled equipment (**B**). Blue ellipses are trials with low scores (≤ 3); Red ellipse are trials with high scores (≥ 7); Black ellipse are trials with very high scores (≥ 9). Trials are color-coded based on score result.

Figure 3 shows the space distribution of shoulder-racket distances at ball impact in each plane for trials with low scores, high scores, and very high scores. The area of the ellipses capturing the distribution of low score trials was greater than the distribution of high score and very high score trials for both groups and in each plane. The full-sized group displayed greater ellipsoid area for low scores than the scaled group in each plane. In both sagittal (side view) and coronal (front view) planes, the orientation between the low score distributions and the very high score distribution is almost perpendicular (see Supplementary Table S5), and this is most evident in the scaled group. Overall, the distribution for very high scores were represented by more elongated ellipses in the scaled group than the full-sized group.

## Discussion

This study advanced our previous findings^11^ relating to children’s motor coordination in a tennis forehand task. We demonstrated that the distance between the shoulder and the racket (i.e., the hitting lever) is a salient performance variable, regardless of the equipment used. We also revealed the contribution of the hand-racket segment to regulating the shoulder-racket distance. Akin to our previous finding, a notable difference between scaled and full-sized equipment was the manner in which children controlled the shoulder-racket distance. Scaled equipment shaped a more distal control, which was the product of functional couplings and de-couplings in the hitting arm’s segments (i.e., the elementary variables). Comparatively, full-sized equipment constrained a more proximal control, and this accompanied by less pronounced couplings amongst the hitting arm’s segments.

Our first step in establishing the relation between shoulder-racket distance and performance was to assess the mean and standard deviation of the distance throughout the swing. The similarities in shoulder-racket distance between scaled and full-sized racket from the initiation of the forward swing to ball impact is noteworthy given that the full-sized racket was 6 inches longer than the scaled racket. Indeed, despite the difference in racket length, the mean distance of both groups at ball impact were essentially identical. Additionally, it was significant that the standard deviation of the shoulder-racket distance was reducing for both groups for the first 75% of the swing. This signals that the shoulder-racket distance was being controlled to achieve a certain fixed-point at ball impact. Of course, this might have been due to mechanistic reasons, with players bringing the racket closer to their body with the full-sized racket to reduce its moment of inertia, thereby aiding velocity generation. The increase in standard deviation closer to ball impact is unsurprising given the task, where subtle adaptations to the racket’s trajectory are required to intercept the ball. It is likely that this increase in variability reflects a shift in synergistic control e.g.^19^, with the hand-racket segment playing a larger role in controlling movement in the final moments e.g.^16^. This also explains the final decrease in variability at ball impact, whereby the elementary variables have formed a synergy for the final moment before ball impact.

We then sought to understand the relationship between shoulder-racket distance and performance. A clear difference emerged, with higher scores featuring longer shoulder-racket distances than lower scores, but only at ball impact. Indeed, it was apparent that success in the task was influenced by the ability to control the shoulder-racket distance to achieve a certain fixed-point at ball impact (i.e., a “sweet spot”), regardless of what the distance was at the start of the forward swing. This is consistent with Bootsma and Wierendgen’s^20^ seminal work investigating the forehand in table tennis, whereby variance in the direction that the bat travelled declined towards ball impact, implying the bat direction was a key performance variable. Significantly, in our study, the shoulder-racket distance achieved by both groups paralleled each other for successful trials. This reflects the invariant nature of the shoulder-racket distance to achieve success in this task and suggests that a shoulder-racket distance “sweet spot” might exist, whereby achieving this distance increases the likelihood of successful outcomes. The meaningfulness of this finding is apparent when we consider the breadth of research investigating striking tasks, such as the forehand, yet lever distance has not been recognized as a performance variable. This might reflect differences in participant skill level, with our study focused on children who were considered beginners, whereas other studies have often focused on skilled athletes e.g.,^12,14,21,22^. Indeed, these studies have identified performance variables including the timing of movements^21^, racket kinematics at ball impact^12,14,16^, and the acceleration of the hitting hand at ball impact^22^. Nonetheless, based on the measures reported in previous studies, it appears that the potential importance of the shoulder-racket distance has been overlooked.

A nontrivial difference in our studies has been the effect of equipment on how children control the shoulder-racket distance when performing a forehand. Previously we reported that the upper arm and forearm worked synergistically when using scaled equipment, whereas full-sized equipment led to a freezing of the upper arm and, consequently, the forearm largely controlled the movement^11^. By including the hand-racket segment in the model for the current study, it was clear that scaled equipment facilitated a more distal control whereas full-sized equipment constrained a more proximal control. More specifically, the hand-racket segment was the main contributor in explaining variance in the shoulder-racket distance throughout the swing when using scaled equipment, and this was accompanied by (a) a coupling of the hand-racket segment and the forearm, and; (b) a coupling of the upper arm and forearm (see Fig. 2D). Notably, for the final 25% of the swing, there was a gradual shift in control strategy, with the upper arm being completely released, and the forearm and hand-racket segment entirely explaining variance in the shoulder-racket distance. This was likely due to a de-coupling between the upper arm and hand-racket segment (see Fig. 2D).

For the full-sized equipment group, the opposite trend emerged. Variance in the shoulder-racket distance was explained primarily by the upper arm angle, which was the product of weaker couplings between the hand-racket segment and forearm, and the upper arm and forearm (see Fig. 2C). This might have been the consequence of the full-sized racket’s larger moment of inertia, with a more proximal control enabling the production of sufficient force to wield the racket. Additionally, there was an abrupt change in control strategy with 25% of the swing remaining, with the hand-racket segment entirely explaining variance in the should-racket distance. Finally, there appeared to be another shift in control strategy for the final 10% of the swing, with the upper arm and forearm playing a greater role. The disjointed nature of the control strategies when using full-sized equipment is congruent with our previous study^11^ and is analogous with the control strategies of novice performers^23^. Hence, the use of full-sized equipment by younger children appears to constrain movement coordination to resemble novice-like performance. The difference between facilitating proximal or distal control of movement in our forehand task is also significant. A more distal control (as observed in the scaled equipment group) is likely to be advantageous given the proximal to distal sequence of joint rotations required to strike a ball^15^. Indeed, distal control is likely to promote a finer control of the racket’s location, which is critical when needing to strike a moving ball and hit it accurately.

Similar to the uncontrolled manifold concept^24^, we also sought to understand the variability of the shoulder-racket vector in state-space. The uncontrolled manifold hypothesis contends that there is *good variability* and *bad variability* in movement systems, with the former describing the variability that exists when joints co-vary to form a synergy and stabilize a performance variable, whereas the latter describes variability that destabilizes a performance variable. We therefore assessed the position of the shoulder-racket vector (relative to the shoulder) in each plane (i.e. state space), as this allowed us to measure the vector’s variability (size and direction). Three findings emerged from this analysis. First, highest scores featured the lowest variability (smallest ellipse area). This confirms the invariant nature of this variable (i.e., the “sweet spot”) for successful performance. Second, the direction of the variability for the lowest scores was orthogonal to the highest scores’ in the sagittal and coronal planes. A closer inspection of the location of the variability revealed that unsuccessful performance was associated with striking the ball too far in front of the body; hence, the angle of the shoulder-racket vector was not successfully controlled to strike the ball at the optimal location. It is noteworthy that the orthogonal direction of variability for the low scores resembles the concept of *bad variability* in the uncontrolled manifold hypothesis e.g.^25^. This means the performer was penalized more when shoulder-racket distance varied along the long-axis ellipse for the lowest scores compared to variance along the long-axis ellipse for the highest scores. Third, the ellipses for the highest scores were skinnier for the scaled equipment group. This infers greater stability of the shoulder-racket distance for successful performance when using scaled equipment compared to full-sized equipment.

These findings relating to variability in the shoulder-racket vector have implications for learning. For instance, consider the following: when children successfully hit the ball to the smallest target (10 points), they received knowledge of results feedback to reinforce the movement produced for that trial. However, the proprioceptive feedback that children received in these trials was different based on the equipment used. For the scaled equipment group, variability in the shoulder-racket vector was clearly lowest for the highest scores. This meant they received invariant proprioceptive feedback for a unique upper body posture (or state) for achieving success. Additionally, scaled equipment afforded the functional (coordinative) work of the body segments to more regularly stabilize the position of the shoulder-racket vector in space. Comparatively, for the full-sized group, variability in the shoulder-racket vector was similar for the high and highest scores, meaning there was substantial overlap in the position of the shoulder-racket vector for these trials. Hence, the feedback that the full-sized group received for achieving the ideal state for the shoulder-racket vector was more varied. This implies that children using full-sized equipment had more difficulty in recognizing what successful shots *felt like*, as successful shots could have been achieved with a similar shoulder-racket vector as less successful shots.

Altogether, we predict at least two outcomes from the results of this study. First, children using inappropriately sized equipment (e.g., full-sized) will find it difficult to learn and control the ideal position of controlled (performance) variables due to unspecific proprioceptive feedback (i.e., too much variance). Second, children using appropriately sized equipment will learn to form functional synergies (i.e., the coupling and de-coupling of joints at the appropriate times) to stabilize salient and invariant performance variables. These predictions are congruent with the growing number of studies demonstrating the beneficial effects of scaling the task and equipment in children’s sports on motor performance and learning^10,11,26–36^; albeit, the current study advances this literature by objectively measuring movement control and coordination.

In sum, this study identified the shoulder-racket distance as being a salient performance variable for children performing the forehand stroke in tennis. Significantly, the manner in which the elementary variables (i.e., the hitting arm joint configurations) controlled the shoulder-racket vector differed based on the equipment used, with scaled equipment promoting functional movement variability and greater distal control. It must be acknowledged, however, that our task emphasized accuracy over speed and this consequently limits our ability to generalize the findings to all forehand strokes. For instance, perhaps children would display greater distal control of the movement when using full-sized equipment when speed is prioritized, given that the proximal to distal sequencing of joints facilitates velocity generation^15^. Additionally, we adopted a basic concept of state-space and used a simple analysis of variance—compared to more advanced analysis methods that are based on the concept of the uncontrolled manifold^24,25^. Nonetheless, the current study offers a framework to guide future hypotheses to better understand the control of abundant degrees of freedom in interceptive striking tasks.

## Methods

Participants were the same as in Buszard et al.^11^. However, it is important to note that the current study is different for three reasons: (1) we are testing a different hypothesis; that is, whether the shoulder-racket distance is a salient performance variable; (2) a limitation of our previous study was that we did not include the hand-racket segment in our model; hence, this study advances our previous insights, and (3) the trials analysed were performed on a different day; thus, the data reported is unique to this study.

### Participants

21 children (14 boys and 7 girls; *mean age* = 7.9 years ± 1.1; *mean height* = 128.8 cm ± 7.2 cm) volunteered to participate in this study. This was 4 participants less than Buszard et al. (2020), which was because the kinematic data of 4 participants was un-analyzable. All children had at least six months experience playing tennis in Tennis Australia’s modified tennis program (“Hot Shots”), but no more than 2 years. All children provided written assent to participate in the study while their parents provided written consent. The study was approved by the Research Ethics Committee at the university where the study was conducted.

### Protocol

Children were stratified randomly into two groups—a scaled equipment group (*n* = 10) and a full-sized equipment group (*n* = 11). The scaled group required participants to use a 21-inch racket and a low compression ball (≈ 25% compression of the standard yellow tennis ball). The full-sized group required participants to use a 27-inch racket and a standard tennis ball.

Children performed a forehand hitting task in an indoor biomechanics laboratory. The aim of the task was to hit the ball as accurately as possibly towards a target located 10 m away. The target was a 0.5 × 0.5 m box, which represented a 10-point shot. Additional boxes were marked outside the central box in 0.5 m bands. For instance, the box immediately surrounding the central target was 1 × 1 m in size, and this represented a 9-point shot. These boxes continued to expand, with a 2-point shot being the smallest box. Any shot that landed outside of the 2-point box, but which travelled over the 0.8 m net (which was located 4 m from the player hitting the ball) was awarded 1 point. Shots that did not travel over the net were awarded 0 points. The size of the court was 6 × 8 m, which is accordance with the International Tennis Federation’s recommendations for 6- to 8-year old children. Balls were fed by the experimenter (TB), who was standing adjacent to the central target. The fed balls were required to land in a 1.0 m × 0.5 m box positioned 1.5 m from the net and participants needed to remain within a 1.2 × 1.2 designated hitting area. The designated hitting area was positioned 0.5 m from the ball-landing box and on the forehand side (i.e., from the midline of the ball-landing box). All children performed the forehand task across two blocks of 20 trials; hence, there were 40 trials in total per participant.

### Data capture

The coordinate systems of the hand, forearm and upper arm of the hitting limb were defined by nine (14-mm) reflective markers. An additional nine markers were also placed over landmarks in a static trial to define the wrist, elbow and shoulder joint centres. The racket was modelled by markers at the butt and tip of the racket (these defined the longitudinal axis) and markers on either side of the racket head (these defined the medio-lateral axis). Full details on the biomechanical model can be found in^11^. As a consequence of the limited surface area on children’s hand, coalescence of three distinct markers presented a major challenge for the optical system. We therefore assumed the hand and the racket to constitute a single segment. Cartesian marker coordinates were recorded using a 100 Hz, 22-camera VICON MX system (VICON Motion Systems, Oxford, UK) and were smoothed using a Butterworth digital filter^37^, with a cut-off frequency of 15 Hz. In the global reference frame, positive x, y and z corresponded to right (parallel to the net), forward (toward the net) and upward, respectively.

**Figure 4:**
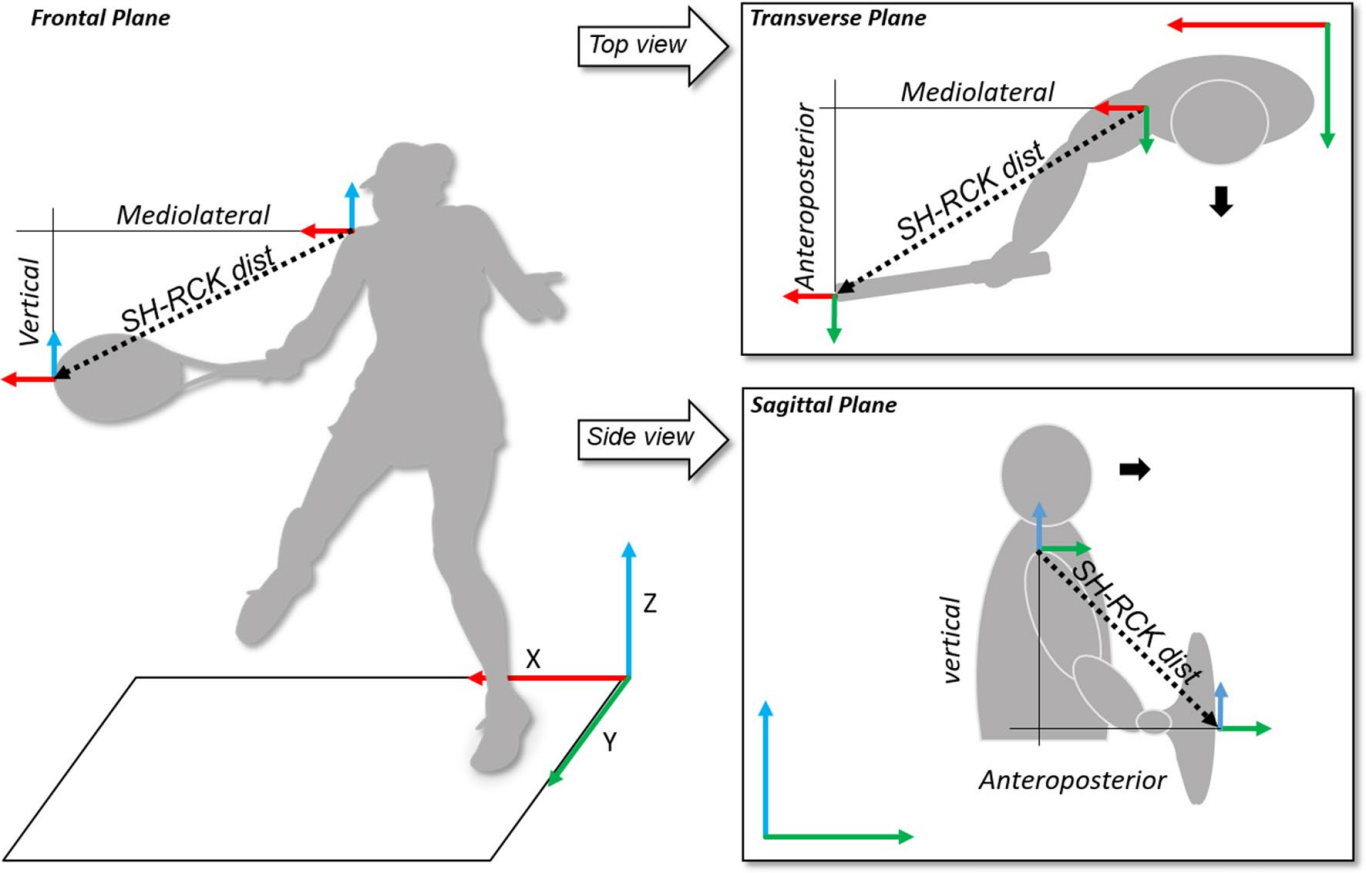
Representation of the shoulder-racket distance in the three planes of state-space.

We defined the start of the forward-swing as when the position (in the y direction) of the racket changed from negative (going backward) to positive (going forward), while the impact frame was identified as the nadir of the unfiltered y-acceleration of the racket tip marker. Transverse plane angles between the hitting arm segments and the racket and their projection on to the ground revealed their alignment. An angle of 0° represented a parallel position of the segment to the ground, while an angle of 90° represented a perpendicular position to the ground. Shoulder-racket distance was the Euclidean distance between the shoulder joint centre and racket tip marker (Fig. 4), which was then normalised by dividing this distance by arm length. Kinematics for the left-handed players were inverted such that all data pertain to a right-hand dominant player.

### Variables of interest

Kinematic variables of interest included shoulder-racket distance and angles of the forearm, upper arm, and the hand-racket segment. Variability in each kinematic variable was calculated between the initiation of the forward-swing and ball impact using the median of absolute deviation (MAD) method^38^. This method calculates the median of the differences between each data point and the circulated median across trials. Variability in shoulder-racket distance was also assessed by visually representing the three components (X–Y–Z) of the shoulder-racket vector at the time of ball impact for each trial based on performance outcome (score). Consequently, the structure of variability in the shoulder-racket vector was assessed in the three planes by fitting a 95% confidence ellipse for trials with low accuracy scores (≤ 3), high accuracy scores (≥ 7), and very accuracy high scores (≥ 9) (frequency of scores are shown in Supplementary Fig. S1). Area, long axis length and angle, short axis length and angle were computed for each ellipse.

### Statistical analysis

Differences in mean shoulder-racket distance for high scores and low scores in both racket conditions at the start of the swing and at ball impact were tested for significance using a two-way ANOVA, with group and score as the factors. The waveforms of the mean shoulder-racket distance in the scaled and full-sized conditions were analysed using one-dimensional statistical parametric mapping (SPM). The effect of group was analysed using a SPM two-tailed repeated measures t-test. The SPM t-test yielded a t-curve, or SPM(t), of which the significance was determined using random field theory^39^. Open source code for conducting SPM tests was obtained (https://www.spm1d.org) and implemented in Matlab version R2018b. Multiple linear regression models were used to determine how shoulder-racket distance variability was explained by variability in the upper arm, forearm, and hand-racket angles in each racket condition. Pearson correlation coefficient (r) was computed for each couple of segments variance (Forearm—Upper arm; Forearm—Hand-Racket; Upper arm—Hand-Racket), and a second order polynomial fit was used to model the distribution of r values over score values. Assumptions of normal distribution and homoscedasticity that underscore ANOVA’s and multiple linear regression analyses were checked via visual inspection of histograms and residual plots. The assumption of *no multicollinearity* for the regression models were checked by inspecting R^2^ values amongst the predictor variables. These values are reported in Supplementary Tables S1 and S2.

The distribution of shoulder-racket vector (categorized by score) at ball impact in the three plane (transversal, sagittal, and coronal) were analysed using the ellipse fit function (Matlab version R2018b). Statistical tests were conducted using SPSS (version 25.0. Armonk, NY: IBM Corp.); statistical significance was set at *p* < 0.05.

### Ethical approval and informed consent

This study was carried out in Accordance with the recommendations of the National Statement on Ethical Conduct in Human Research (2007). All participants gave informed assent and written informed consent was provided by their parents or guardians in accordance with the National Statement. The protocol was approved by the Victoria University Human Research Ethics Committee.

## Supplementary information

Supplementary Information.

## Data Availability

The datasets generated during and/or analysed during the current study are available from the corresponding author on reasonable request.
