# Supplementary material for: Children’s coordination of the “sweet spot” when striking a forehand is shaped by the equipment used
Source: Sci Rep. 2020 Dec 3;10:21003. doi: 10.1038/s41598-020-77627-5 (PMC7713293; doi:10.1038/s41598-020-77627-5)
Supplement: Supplementary file 1 — Supplementary Information. [file 41598_2020_77627_MOESM1_ESM.pdf]

Supplementary Material

Children's coordination of the "sweet spot" when striking a forehand is shaped by the equipment used

Tim Buszard, Alessandro Garofolini, David Whiteside, Damian Farrow & Machar Reid

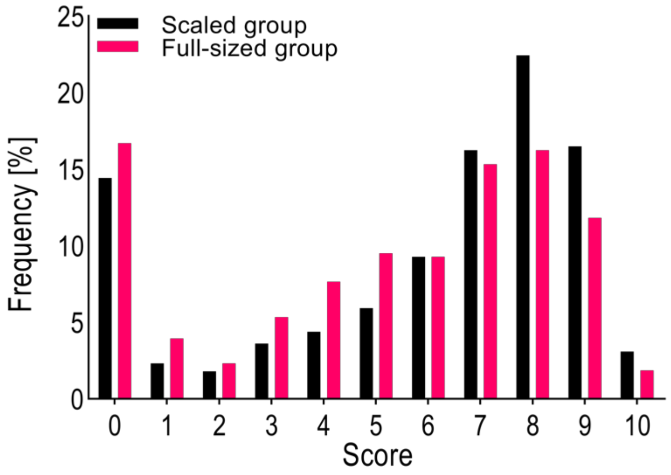

Supplementary Figure 1. Frequency distribution of scores for the two groups.

Supplementary Table 1. Statistical output for the multiple linear regression in the *scaled condition*

**Model***					
Parameter estimates	*Variable*	*Estimate*	*Standard error*	*95% confidence interval*	
β0	Intercept	0.05	0.02	-0.0009 to 0.09	
β1	B : UpperArm	0.008	0.0003	0.007 to 0.009	
β2	C : Forearm	0.02	0.0003	0.017 to 0.018	
β3	D : Racket	0.01	0.0003	0.012 to 0.013	
Sig. diff. than zero?	*Variable*	|*t*|	*P value*	*P value summary*	
β0	Intercept	1.920	0.0548	ns	
β1	B : UpperArm	24.08	<0.0001	****	
β2	C : Forearm	53.29	<0.0001	****	
β3	D : Racket	44.61	<0.0001	****	
Goodness of Fit					
Degrees of Freedom	19588				
R squared	0.27				
Analysis of Variance	*SS*	*DF*	*MS*	*F (DFn, DFd)*	*P value*
Regression	2523	3	840.9	F (3, 19588) = 2460	P<0.0001
Residual	6696	19588	0.34		
Total	9219	19591			
Multicollinearity	*Variable*	*VIF*	*R^2 with other variables*		
β0	Intercept				
β1	B : UpperArm	1.01	0.009		
β2	C : Forearm	1.12	0.10		
β3	D : Racket	1.12	0.11		
Correlation matrix	*Variable*	*β0*	*β1*	*β2*	*β3*
β0	Intercept	1.00			
β1	B : UpperArm	-0.91	1.00		
β2	C : Forearm	-0.26	0.02	1.00	
β3	D : Racket	-0.25	0.08	-0.32	1.00

*A least squares regression model; dependent variable = shoulder-racket distance; formula = Y = β0 + β1*B + β2*C + β3*D

Supplementary Table 2. Statistical output for the multiple linear regression in the *full-sized condition*

**Model***					
Parameter estimates	*Variable*	*Estimate*	*Standard error*	*95% confidence interval*	
β0	Intercept	0.02	0.02	-0.02 to 0.07	
β1	B : UpperArm	0.02	0.0003	0.02 to 0.02	
β2	C : ForeArm	-0.007	0.0004	-0.008 to -0.006	
β3	D : Racket	0.005	0.0004	0.004 to 0.006	
Sig. diff. than zero?	*Variable*	$\|t\|$	*P value*	*P value summary*	
β0	Intercept	1.04	0.30	ns	
β1	B : UpperArm	55.45	<0.0001	****	
β2	C : Forearm	19.06	<0.0001	****	
β3	D : Racket	12.35	<0.0001	****	
Goodness of Fit					
Degrees of Freedom	21811				
R squared	0.16				
Analysis of Variance	*SS*	*DF*	*MS*	*F (DFn, DFd)*	*P value*
Regression	2087	3	695.8	F (3, 21811) = 1442	P<0.0001
Residual	10523	21811	0.48		
Total	12611	21814			
Multicollinearity	*Variable*	*VIF*	*R^2 with other variables*		
β0	Intercept				
β1	B : UpperArm	1.07	0.06		
β2	C : Forearm	1.07	0.06		
β3	D : Racket	1.08	0.07		
Correlation matrix	*Variable*	*β0*	*β1*	*β2*	*β3*
β0	Intercept	1.00			
β1	B : UpperArm	-0.91	1.00		
β2	C : Forearm	-0.40	0.18	1.00	
β3	D : Racket	-0.04	-0.20	-0.21	1.00

*A least squares regression model; dependent variable = shoulder-racket distance; formula = Y = β0 + β1*B + β2*C + β3*D

Supplementary Table 3. Statistical output for the second order polynomial in the *scaled condition*

**Second order polynomial (quadratic)**	**FRvsUP***	**FRvsRK***	**UPvsRK***
Best-fit values			
B0	-0.86	0.48	-0.72
B1	0.45	0.007	0.09
B2	-0.04	0.003	-0.002
95% CI (profile likelihood)			
B0	-1.51 to -0.21	-0.51 to 1.46	-0.99 to -0.44
B1	0.15 to 0.76	-0.45 to 0.46	-0.04 to 0.29
B2	-0.07 to -0.01	-0.04 to 0.05	-0.01 to 0.01
Goodness of Fit			
Degrees of Freedom	8	8	8
R squared	0.60	0.05	0.73
Sum of Squares	1.10	2.51	0.20
Sy.x	0.37	0.56	0.16
Number of points			
# of X values	11	11	11
# Y values analysed	11	11	11

*FR = Forearm angle; UP = upper arm angle; RK = racket angle.

Supplementary Table 4. Statistical output for the second order polynomial in the *full-sized condition*

Second order polynomial (quadratic)	FRvsUP*	FRvsRK*	UPvsRK*
Best-fit values			
B0	-0.18	0.12	-0.51
B1	0.20	-0.20	-0.03
B2	-0.02	0.02	0.006
95% CI (profile likelihood)			
B0	-1.40 to 1.04	-0.62 to 0.85	-1.56 to 0.54
B1	-0.37 to 0.76	-0.54 to 0.14	-0.51 to 0.46
B2	-0.07 to 0.03	-0.007 to 0.06	-0.04 to 0.05
Goodness of Fit			
Degrees of Freedom	8	8	8
R squared	0.07	0.40	0.05
Sum of Squares	3.86	1.40	2.85
Sy.x	0.69	0.42	0.60
Number of points			
# of X values	11	11	11
# Y values analyzed	11	11	11

*FR = Forearm angle; UP = upper arm angle; RK = racket angle.

Supplementary Table 5. Space distribution of shoulder-racket distances at ball impact in each plane for trials with low scores (≤ 3), high scores (≥ 7), and very high scores (≥ 9).

	**Full-sized**			**Scaled**		
	Low	*High*	*Very High*	*Low*	*High*	*Very High*
Transverse Plane						
Ellipse Area (a.u.)	2.65	0.84	0.6	1.58	1.18	0.58
Long axis						
length (a.u.)	3.25	1.47	1.18	2.42	2.14	1.13
angle (deg)	131	125	119	127	126	148
Minor axis						
length (a.u.)	1.04	0.73	0.65	0.83	0.7	0.65
angle (deg)	41	35	29	37	36	58
Sagittal Plane						
Ellipse Area (a.u.)	3.31	1.42	1.17	2.51	1.8	0.64
Long axis						
length (a.u.)	2.28	1.81	1.59	2.4	1.56	1.36
angle (deg)	36	124	111	132	71	116
Minor axis						
length (a.u.)	1.85	1	0.94	1.33	1.47	0.6
angle (deg)	-54	34	21	42	-19	26
Coronal Plane						
Ellipse Area (a.u.)	3.29	1.2	0.88	2.14	1.49	0.7
Long axis						
length (a.u.)	2.38	1.84	1.5	2.02	1.61	1.66
angle (deg)	155	63	84	126	59	52
Minor axis						
length (a.u.)	1.76	0.83	0.75	1.35	1.18	0.54
angle (deg)	65	-27	-6	36	-31	-38
